# Zebrafish Suppressor of Cytokine Signaling 4b (Socs4b) Is Dispensable for Development but May Regulate Epidermal Growth Factor Receptor Signaling

**DOI:** 10.3390/biom14091063

**Published:** 2024-08-26

**Authors:** Monique Trengove, Parisa Rasighaemi, Clifford Liongue, Alister C. Ward

**Affiliations:** 1 School of Medicine, Deakin University, Geelong, VIC 3216, Australiac.liongue@deakin.edu.au (C.L.); 2 Institute for Mental and Physical Health and Clinical Translation, Deakin University, Geelong, VIC 3216, Australia

**Keywords:** cytokine, EGFR, SOCS, SOCS4

## Abstract

The suppressor of cytokine signaling (SOCS) family of proteins were named after their defining role as negative feedback regulators of signaling initiated by numerous cytokine receptors. However, multiple members of the SOCS family likely function outside of this paradigm, including SOCS4. Zebrafish possess two *SOCS4* paralogues, with *socs4a* previously shown to participate in central nervous system development and function. This study examined the role of the other paralogue, *socs4b*, through expression analysis and functional investigations in vivo and in vitro. This revealed maternal deposition of *socs4b* mRNA, specific zygotic expression during late embryogenesis, including in the brain, eye and intestine, and broad adult expression that was highest in the brain. A mutant allele, *socs4bΔ18*, was generated by genome editing, in which the start codon was deleted. Fish homozygous for this likely hypomorphic allele showed no overt developmental phenotypes. However, in vitro studies suggested the Socs4b protein may be able to regulate EGFR signaling.

## 1. Introduction

The suppressor of cytokine signaling (SOCS) family of proteins were initially identified as inducible negative regulators of signaling stimulated by cytokine receptors [1]. In this manner, the SOCS proteins serve to ensure that these downstream pathways are extinguished in a controlled and appropriate way [2]. Subsequent studies have suggested that this paradigm predominates for the subset consisting of SOCS1, SOCS2, and SOCS3, as well as the idiosyncratically termed cytokine-inducible SH2-containing protein (CISH), which have evolved more recently in parallel with the emergence and expansion of cytokines and their receptors [3]. This subset of SOCS proteins are especially important in the regulation of blood and immune cell development and function, but also exert other homeostatic roles [4]. Thus, SOCS1 is particularly critical in controlling interferon (IFN) signaling, mitigating its pathogenic effects [5]. SOCS2 plays a key role in regulating growth hormone (GH) signaling, impacting growth and adiposity [6,7]. SOCS3 is an essential physiological regulator of granulocyte colony-stimulating factor (G-CSF) and other cytokines [8,9,10] that impact myeloid cell development and function as well as additional cytokines, such as leukemia inhibitory factor (LIF) and leptin (LEP), to impact diverse developmental and homeostatic processes [11,12]. CISH exerts itself at multiple levels, including controlling interleukin 4 (IL-4) and T-cell receptor (TCR)-mediated T cell development [13,14], interleukin 15 (IL-15)-mediated signaling in natural killer (NK) cells [15], basal and granulocyte-macrophage colony-stimulating factor (GM-CSF)-mediated myelopoiesis [16], basal and erythropoietin (EPO)-induced erythropoiesis [17], and LEP-mediated appetite control [18].

In contrast, SOCS4, SOCS5, SOCS6, and SOCS7 have a longer evolutionary history [3,19] and participate more strongly in the regulation of growth factor receptor signaling and other pathways [2]. Thus, SOCS6 has been shown to impact signaling via insulin receptor substrate 4 (IRS4) to regulate growth [20], while SOCS7 regulates reelin signaling to impact cortical neuron migration [21]. Both SOCS4 and the related SOCS5 have been implicated in regulating signaling by the epidermal growth factor (EGF) receptor [22], but understanding of their physiological roles remains very limited.

The single mammalian SOCS4 has been shown to be broadly expressed [23,24], and able to be regulated by EGF stimulation [22] and viral infection [25] at the gene and protein levels, respectively. However, ablation of the mouse *Socs4* gene failed to elicit an overt phenotype, but resulted in increased susceptibility to the pathogenic effects of virus infections [24,26], with similar results obtained when the mouse *Socs5* gene was ablated [27,28]. A *SOCS4* mutation was also associated with autoimmunity in a human pedigree [29]. In addition, potential functions for SOCS4 in regulating wound healing [30] and primordial follicle activation [31] have been suggested.

The zebrafish represents an exquisite alternative model for the study of gene function in development and its perturbation [32]. Like other teleost fish, it possesses one or more copies of all eight mammalian *SOCS* genes, including two *SOCS4* paralogous, *socs4a* and *socs4b* [3], and has proved informative for the study of paralogues of mammalian SOCS1 [33,34], SOCS3 [10], and CISH [35]. This has recently been extended to the investigation of *socs4a*, which identified that this gene functions in notochord development and formation of a functional sensory system during zebrafish embryogenesis [36]. In this study, the role of the zebrafish *socs4b* paralogue was investigated through expression analysis and functional investigations, including the generation and characterization of a *socs4b* mutant generated by genome editing, complemented by in vitro biochemical studies. This revealed that *socs4b* was dispensable for development despite broad expression but may regulate EGFR signaling.

## 2. Materials and Methods

### 2.1. Zebrafish Husbandy and Manipulation

Zebrafish were maintained using standard husbandry methods in an Aquatic Habitats aquarium facility. This functioned on a 14 h light/10 h dark light-cycle, with fish fed three times daily on a diet that included brine shrimp and high protein pellets. Spawning was stimulated at the beginning of the light cycle with the resultant embryos manually collected and placed in egg-water containing 3 ppm methylene blue (Sigma-Aldrich, Melbourne, VIC, Australia) and allowed to develop at 28.5 °C. RNAs encoding a pair of zinc-finger nuclease (ZFN) proteins that targeted the sequences around the *socs4b* start codon were provided by a commercial supplier (Sigma). These were diluted to 150 pg/nL in 1× Danieau containing 1% (*w*/*v*) phenol red and injected into 1–8 cell stage embryos held with watchmakers’ forceps using needles pulled from filamented 1.0 mm glass capillary tubes (SDR Scientific Pty Ltd., Chatswood, NSW, Australia) mounted into a MN-151 micromanipulator (Narishige International, London, United Kingdom) attached to a SMZ 645 dissecting microscope (Nikon Australia, Rhodes, NSW, Australia). The injected embryos were raised to adulthood, with identified heterozygote mutant fish out-crossed three times to wild-type (WT) fish before being in-crossed to generate homozygote mutants.

### 2.2. Genomic Analysis

Genomic DNA (gDNA) was prepared from whole embryos or adult fin clips using QuickExtract DNA Extraction Solution (Epicentre) by sequential vortexing for 30 s, incubation at 68 °C for 2 min, vortexing for 30 s, incubation at 98 °C for 2 min, and vortexing for 30 s before centrifugation, with the supernatant used in subsequent PCR assays using the primers: 5′-CGTTATGAGCACCTTTGACTGA and 5′-ATCTTCTGTCGGAGTGTGCGG. This included initial screening for mutations using a Surveyor Mutation Detection Kit (Transgenomic Inc, New Haven, CT, USA) following the manufacturers’ guidelines in each case. Digested PCR products were analyzed by polyacrylamide gel electrophoresis (PAGE) on 10–30% [29:1 acryl:bis-acryl] acrylamide gels, containing 1× TBE buffer (89 mM Tris borate, 2 mM EDTA pH 8.0) and run in a minigel apparatus (BioRad Laboratories, South Granville, NSW, Australia) at 100 V for 60 min. Gels were post-soaked in Sybrsafe (1:10,000) (Invitrogen Australia, Mount Waverley, VIC, Australia) and visualized under UV illumination (excitation 480 nm, emission 620 nm) on a ChemiDoc XRS (BioRad). Alternatively, PCR products were sequenced by the Australian Genome Research Facility (AGFR) utilizing BigDye Terminator sequencing and capillary separation on a 3730xl 96-capillary sequencer (Applied Biosystems, Thermo Fisher Scientific Pty Ltd., Scoresby, VIC, Australia).

### 2.3. Whole-Mount In Situ Hybridization (WISH) and Neuromast Staining

The embryos were raised in petri dishes using egg-water containing 0.003% (*w*/*v*) 1-phenyl-2-thiourea (PTU) from 9 h post-fertilization (hpf) to inhibit pigment formation. At appropriate times, the embryos were euthanized using 100 mg/L benzocaine (Sigma) prior to fixation in 4% (*w*/*v*) paraformaldehyde (PFA) in phosphate-buffered saline (PBS) (137 mM NaCl, 2.7 mM KCl, 4.3 mM Na_2_HPO_4_, 1.47 mM KH_2_PO_4_) (PFA/PBS) before dehydration with 100% (*v*/*v*) methanol for long-term storage at −20 °C. Rehydrated embryos were subjected to WISH using Digoxygenin (DIG)-labelled RNA probes [37], with some embryos sectioned and counter-stained with FastRed. Alternatively, neuromasts were visualized in anesthetized embryos using MitoTracker Red CMX Ros (Invitrogen). Embryos were imaged under stage lighting or fluorescence as required using an SZX-12 microscope coupled with a DP70 camera using DP Controller software v3.3.1 (Olympus, Shinjuku, Japan).

### 2.4. Reverse Transcription–Polymerase Chain Reaction (RT-PCR)

The total RNA was isolated from zebrafish embryos or microdissected adult tissues using TRIzol reagent (Invitrogen), following the manufacturer’s guidelines. Semi-quantitative RT-PCR was performed using primers specific for *socs4b* (full-length: 5′-ACTGAGTGAGTGTGGTCTGA and 5′-CGGATGTAAAGGCACTGGT; N-terminus: 5′-ACTGAGTGAGTGTGGTCTGA and 5′-GGTCTGCTGGAGGCTGTCTG; and C-terminus: 5′-GCTCCGTCCGTCATCCGA and 5′-CGGATGTAAAGGCACTGGT), and *actb* (5′-AACACAACACAGGATCATGGAG; 5′-CATTGCTACACTTGCTTCTTGC). Products were separated by electrophoretic analysis performed on 1% (*w*/*v*) agarose gels containing 1× Tris-acetate-EDTA (TAE) buffer (40 mM Tris-HCl, 190 mM glacial acetic acid, 10 mM EDTA; pH 8.0) (BioRad) and Sybrsafe (Invitrogen) (1:10000) in a mini-tank apparatus (BioRad) at 100-110 V for 30-80 min, as appropriate, and visualized on a ChemiDoc XRS. Quantitative real-time RT-PCR (qRT-PCR) was performed on an Mx3000P qPCR machine (Agilent Technology Australia, Mulgrave, VIC, Australia) with these primers, as well as those for *socs4a* (5′-GGAGCGGACGAACAGACT; 5′-AGATGCCAGCACTGAGCG), *socs5a* (5′-GATTATCGCAGTTATGTTCCC and 5′-GTGGCATACCGTGTCTGTTTG), and *socs5b* (5′-GGGTAATCTTAGAAGCCGA and 5′-TGAGGAATATCCGCCACA). Data were extracted using MxPro qPCR software v4.10 (Stratagene), normalized to *actb*, and the fold change calculated using the ΔΔCt method [38].

### 2.5. In Vitro Transcription/Translation

Sequences encoding Socs4b variants were amplified from gDNA obtained from *socs4b* WT and *socs4bΔ18* fish using specific flanking primers (5′-ACTGAGTGAGTGTGGTCTGA and 5′-CGGATGTAAAGGCACTGGT) and cloned into pGEM2T (Invitrogen) for in vitro transcription/translation. The pGEM2T plasmids harboring *socs4b* WT and *socs4bΔ18* were used in a TNT Quick Coupled Transcription/Translation System reaction along with a no template control, with the products analyzed by SDS-PAGE as described previously [35].

### 2.6. Biochemical Studies

Alternative primers were used to generate Flag-tagged versions of full-length Socs4b (Socs4b WT) (5′-GCGAAGCTTAGCCTCATCATGTCTCTG and 5′-CTGAATTCCGGATGTAAAGGCACTGGT) and a variant commencing at the second in-frame methionine (Socs4b Δ18) (5′-GCGAAGCTTATGCTGGACCGCTGCCCCTTC and 5′-CTGAATTCCGGATGTAAAGGCACTGGT) from WT fish, with products cloned into the *Hin*dIII/*Eco*RI sites of p3xFlag-CMV-10 (Sigma). HEK293T cells in 6-well plates were transiently transfected with these plasmids along with an empty vector control using Fugene HD reagent (Roche) and incubated at 37 °C in 10% (*v*/*v*) CO_2_ for 46–48 h prior to stimulation by 20 µg/mL of epidermal growth factor (EGF) (Sigma). Cell lysates were collected at 48 h post transfection for Western blot analysis. The membranes were blocked for 1 h at room temperature in 5% (*w*/*v*) bovine serum albumin (BSA) (Sigma) in Tris-buffered saline-Tween (TBST) (122 mM Tris pH 7.6, 900 mM NaCl, 0.001% (*v*/*v*) Tween-20), followed by incubation in 1:200 mouse anti-total EGFR (sc-53274; Santa Cruz), 1:800 rabbit anti-phospho-EGFR-Y845 (sc-23420-R; Santa Cruz), 1:1000 rabbit anti-total ERK1/2 (#9102; Cell Signaling), 1:1000 rabbit anti-phospho-ERK1/2-T202/Y204 (#9101; Cell Signaling), 1:2000 rabbit anti-Flag (#SAB4301135; Sigma-Aldrich), 1:10000 anti-GAPDH (#PA1-988; LifeTechnologies) in 5% (*w*/*v*) of BSA/TBST overnight at 4 °C with gentle agitation. The membranes were subsequently washed three times in TBST for 10 min and then incubated with 1:10000 secondary antibody, IRDye 800CW Goat anti-Mouse IgG (#926-32210; LI-COR) or IRDye 680 Goat anti-Rabbit IgG (#926-68071; LI-COR), in 5% (*w*/*v*) of BSA/TBST for 1 h at RT. Unbound antibodies were then removed by washing the membranes twice in TBST for 15 min. Antibody binding was detected and imaged using an Odyssey Imager (LI-COR, Millenium Science, Mulgrave, VIC, Australia).

### 2.7. Quantitation and Statistical Analysis

Data were analyzed with a Student’s *t*-test with Welch’s correction as required, utilizing GraphPad Prism 8.0, with *p* < 0.05 considered significant. Where appropriate, bands were quantified with ImageJ software v1.41 and standardized against relevant loading controls.

## 3. Results

### 3.1. Expression of Socs4b

**Figure 1:**
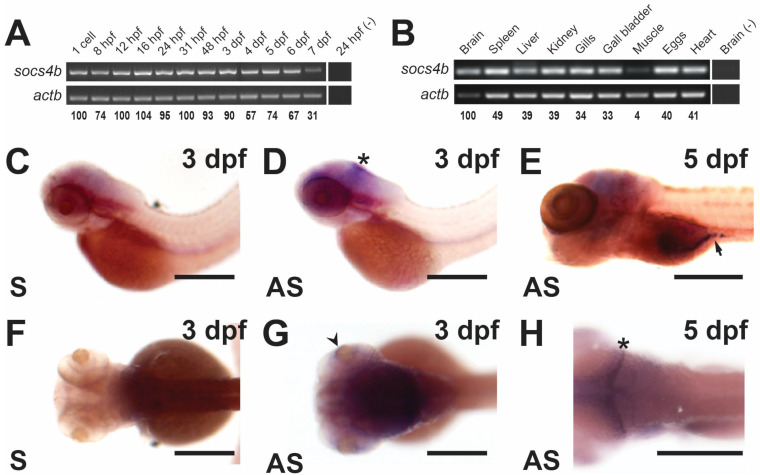
Spatio-temporal expression profile of the zebrafish *socs4b* gene. Semi-quantitative RT-PCR analysis of *socs4b* and the control *actb* gene using total RNA extracted from embryos at the indicated times post-fertilization (**A**) or from the indicated microdissected adult tissues (**B**). Individual bands were quantified to calculate the relative ratio of *socs4b*:*actb* that is shown below each panel, with the first sample set at 100% in each case. Whole-mount in situ hybridization analysis of *socs4b* on embryos at 3 dpf (**C**,**D**,**F**,**G**) and 5 dpf (**E**,**H**) using either sense (S) or anti-sense (AS) *socs4b* probes, as indicated, imaged either laterally (**C**–**E**) or dorsally (**F**–**H**). Asterisks indicate staining in the brain in panels (**D**,**H**), the arrowhead indicates staining in the retina in panel (**G**), and the arrow staining in the intestine in panel (**E**). Scale bars = 0.5 mm.

To provide insight into possible functionality of the zebrafish *socs4b* gene, its embryonic and adult expression was analyzed. Temporal expression during embryogenesis was first assessed using semi-quantitative RT-PCR with gene-specific primers designed to the first half of the coding region, covering the N-terminal conserved region (NTCR)4-5 and part of the SH2 domain (Supplementary Figure S1). This analysis revealed that *socs4b* transcripts were already present at the 1 cell stage, indicating maternal deposition, with levels dipping slightly at 8 h post-fertilization (hpf) before returning to similar levels from 12 hpf to 3 days post-fertilization (dpf) and then declining through to 7 dpf (Figure 1A). In contrast, the expression levels of the housekeeping gene beta-actin (*actb*) were consistent across all time-points. Embryonic expression was further investigated with WISH using a full-length anti-sense *socs4b* RNA probe, with a corresponding sense probe used as a control. Specific staining was observed with the anti-sense probe from 3 dpf in the brain (Figure 1C,D,H), as well as the eye (Figure 1F,G), with expression also observed in the intestine at 5 dpf (Figure 1E) and beyond. Analysis of multiple adult tissues using RT-PCR revealed broad expression of *socs4b*, with the highest relative levels in the brain and the lowest relative levels in skeletal muscle (Figure 1B).

### 3.2. Targeted Mutation of Zebrafish socs4b

**Figure 2:**
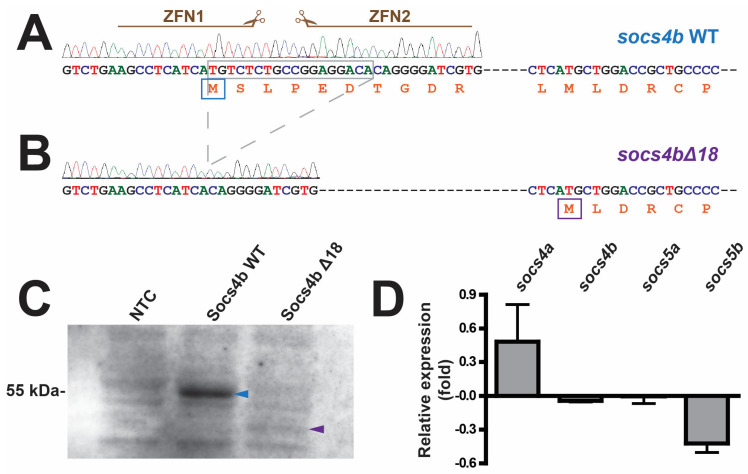
Zinc-finger nuclease-mediated targeting of the zebrafish *socs4b* gene and evaluation of the *socs4bΔ18* mutation. Sequence of zebrafish *socs4b* WT (**A**) and *socs4bΔ18* (**B**) alleles, including the corresponding chromatograms, with the sequences targeted by the zinc-finger nuclease (ZFN) pair indicated and the nucleotides deleted in the *socs4bΔ18* mutant shown by the grey box. Hyphens represent 258 nucleotides of intervening sequences prior to the first in-frame methionine. The respective protein translations are presented below, with the native start methionine of Socs4b WT indicated by a blue box and the next in-frame methionine in Socs4bΔ18 with a purple box. In vitro transcription/translation analysis of Socs4b WT and Socs4bΔ18 sequences along with a no template control (NTC) (**C**). The blue arrowhead indicates full-length Socs4b WT protein product and the purple arrowhead indicates the truncated Socs4bΔ18 protein product. Quantitative RT-PCR analysis of the *socs4a*, *socs4b*, *socs5a*, and *socs5b* genes performed on total RNA extracted from *socs4b* WT and *socs4bΔ18* embryos, expressed as a fold-change *socs4bΔ18* relative to *socs4b* WT embryos, with error bars indicating the standard error of the mean (**D**). Statistical analysis determined all differences as *p* > 0.05. Original blot can be found in Supplementary Figure S2.

To directly investigate the function of the zebrafish *socs4b* gene, it was targeted for mutation using zinc-finger nuclease (ZFN) mediated genome editing [39]. In vitro transcribed RNAs encoding a pair of ZFNs targeting area 6 base pairs (bp) downstream of the start codon (Figure 2A, Supplementary Figure S1A) were injected into single cell wild-type (WT) embryos. These were raised to adulthood and subsequently mated with WT fish, with their offspring screened for potential germ line *socs4b* mutations via the Cel I method [40]. Putative mutants were confirmed by the sequencing of homozygotes, which identified an allele containing an 18 bp deletion that included the *socs4b* start codon, termed *socs4bΔ18* (Figure 2B). The next available in-frame methionine lies 130 amino acids downstream of the start site and approximately halfway into the N-terminal conserved region of SOCS4/5 (NTCR4-5) domain (Supplementary Figure S1C).

To better understand the impacts of the introduced mutation, plasmids containing either the *socs4b* WT or *socs4bΔ18* sequence were subjected to in vitro transcription/translation with the resultant protein products separated by gel electrophoresis (Figure 2C). The *socs4b* WT produced a protein at the expected size of ~55 kDa while the *socs4bΔ18* template generated no product at this size, but instead yielded a small amount of a protein at ~41 kDa, close to the predicted size for a product generated from the second in-frame methionine located 130 residues downstream, suggesting this methionine is utilized, albeit poorly.

### 3.3. Phenotypic Analysis of Socs4b Mutants

Inheritance of the *socs4bΔ18* allele was found to follow classical Mendelian inheritance, with homozygote mutants being robust and fertile, enabling the establishment of a homozygote *socs4bΔ18* line (Supplementary Figure S3A), with a WT line derived from littermates. Embryos from each line were subjected to RT-PCR analysis using primer sets designed to the regions of *socs4b* encoding the N-terminus and C-terminus of the protein (Supplementary Figure S1B and S3B). In each case, products of similar intensity were produced from homozygous *socs4b* WT and *socs4bΔ18* embryos, but the products were shorter with the N-terminal primers in *socs4bΔ18* mutants compared to *socs4b* WT embryos, which was not the case for the products of the C-terminal primers. This demonstrates that the truncation was incorporated into the mRNA transcript and without impacting expression. Quantitative RT-PCR analysis was also performed on *socs4b* in concert with the *socs4a* paralogue, as well as the related *socs5* paralogues, *socs5a* and *socs5b*. No significant changes were observed for any of these genes between *socs4b* WT and *socs4bΔ18* embryos at either 24 hpf (Figure 2D) and confirmed at 7 dpf, suggesting an absence of compensation.

**Figure 3:**
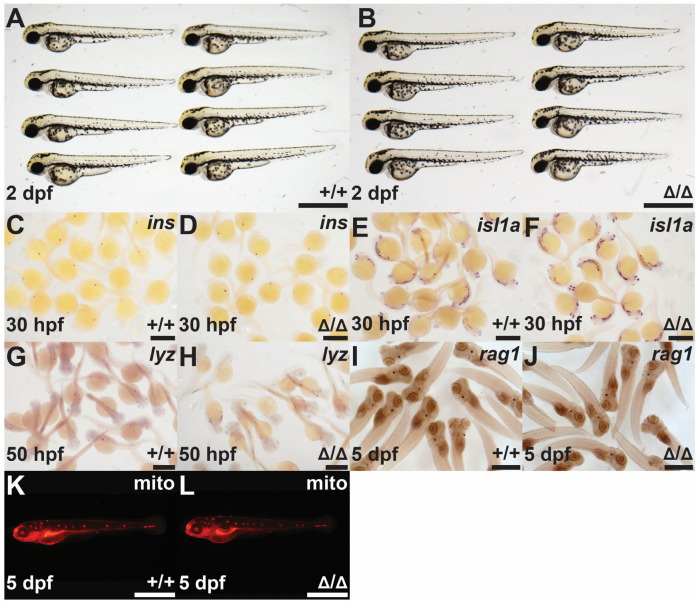
Investigation of the *socs4bΔ18* mutants. Light microscopy of homozygous *socs4b* WT (+/+; (**A**)) and *socs4bΔ18* (Δ/Δ; (**B**)) embryos at 2 dpf. Homozygous *socs4b* WT (+/+; (**C**,**E**,**G**,**I**,**K**)) and *socs4bΔ18* (Δ/Δ; (**D**,**F**,**H**,**J**,**L**)) embryos subjected to WISH at the indicated times with specific probes for *ins* ((**C**), n = 35; (**D**), n = 33), *isl1a* ((**E**), n = 42; (**F**), n = 38), *lyz* ((**G**), n = 27; (**H**), n = 30), and *rag1* ((**I**), n = 40; (**J**), n = 37) viewed by light microscopy or staining with Mitotracker Red (mito) ((**K**), n = 23; (**L**), n = 25) and viewed by fluorescence microscopy. The number of embryos analyzed with each stain is shown. Scale bars = 1 mm.

The embryos were carefully assessed by light microscopy from 1 cell to 7 dpf, but no gross developmental abnormalities were observed (Figure 3A,B). To further investigate potential phenotypes in the mutants, WISH analysis was performed using markers for key cell populations during development. These genes included insulin (*ins*), marking the β cells of the developing endocrine pancreas [41]; islet 1a (*isl1a*), marking central nervous system (CNS) anatomy, including sensory motor neurons [42]; lysozyme (*lyz*), marking leucocytes [43]; and recombination activation gene 1 (*rag1*), marking early T lymphocytes [44], with Mitotracker Red staining as a mitochondrial stain to highlight lateral line neuromasts [45]. These analyses revealed no abnormal number or distribution of any of the marked cell populations in *socs4bΔ18* embryos when compared to WT controls (Figure 3C–L).

### 3.4. Regulation of EGFR Signaling by Socs4b

**Figure 4:**
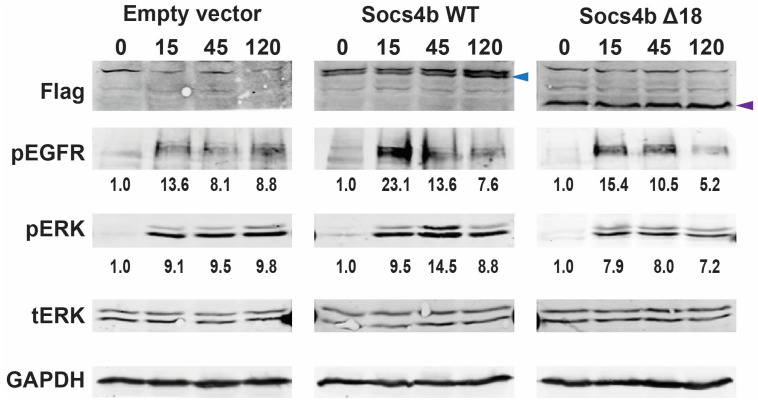
Analysis of the impact of zebrafish Socs4b on EGFR signaling. HEK293T cells transfected with empty vector control or plasmids encoding Flag-tagged Socs4b WT or Socs4b Δ18 were stimulated with EGF for the time indicated (min). Protein was extracted and subjected to Western blot analysis with antibodies against the Flag-tag to confirm expression of the Socs4b forms, as well as to pEGFR and pERK to detect EGFR signaling, along with tERK and glyceraldehyde 3-phosphate dehydrogenase (GAPDH) that served as loading controls. The position of Flag-tagged Socs4b WT and Socs4b Δ18 proteins are shown with blue and purple arrowheads, respectively. Bands were quantified and the relative ratio of pEGFR:GAPDH and pERK:tERK calculated and shown below the relevant panel, with the 0 min sample set at 1.0 in each case, showing the average of two independent replicates. Original blots can be found in Supplementary Figure S4.

SOCS4 has been previously implicated in EGF receptor (EGFR) signaling [46], and so the potential involvement of zebrafish Socs4b in EGFR signaling was investigated in vitro. The expression vector pBK-CMV, and versions containing sequences encoding Flag-tagged Socs4b WT or Socs4b Δ18 were transfected into HEK293T cells, which express EGFR endogenously. The expression of the two Flag-tagged Socs4b constructs was confirmed, with products at the expected sizes of ~56 kDa (Flag-Socs4b WT) and ~42 kDa (Flag-Socs4b Δ18) (Figure 4). Stimulation of cells transfected with the empty vector using EGF resulted in the strong phosphorylation of EGFR, as well as extracellular-regulated kinase (ERK), which lies downstream of EGFR, from 15 min to 2 h. Cells transfected with Socs4b WT showed enhanced levels of pEGFR at 15 min and 45 min, as well as pERK at 45 min. In contrast, Socs4b Δ18 samples showed similar pEGFR and pERK levels to the empty vector transfected samples.

## 4. Discussion

The SOCS family of proteins represent a major mode of regulation for signaling via cytokines and other factors, particularly impacting blood and immune cell development and function, but with many other developmental and homeostatic roles [1,4]. This study sought to address the paucity of information regarding the function of one member of this family, SOCS4. This was achieved through the use of zebrafish as a developmental model to investigate the role of its *socs4b* paralogue, employing expression analysis, phenotypic investigation of a genome-edited mutant, buttressed with in vitro studies.

The embryonic expression pattern of mammalian *Socs4* has not been described previously, and so analysis of *socs4b* expression during embryogenesis was able to provide new insights. Transcripts for this gene were found to be maternally derived and maintained at a relatively consistent level throughout embryogenesis, with specific expression in the brain, at what appears to be the boundary of the third and fourth ventricles, as well as in the developing eye, likely in the retina, as well as the intestine. This contrasted with the zebrafish *socs4a* gene paralogue, which displayed initial ubiquitous expression before becoming restricted to the sensory ganglion [36]. However, as was the case with *socs4a*, some locations of *socs4b* mRNA expression overlapped with sites of expression of the zebrafish *stat3* gene. In particular, zebrafish *stat3* has also been shown to be expressed in the cells lining the ventricles of the brain and the retina [47], although expression of *stat3* at this location was more extensive than that seen with *socs4b*. This overlapping expression suggests potential interaction between these genes in some way, although much additional analysis would be required to fully investigate this relationship. Mammalian *Socs4* has been shown to be expressed in the adult olfactory bulb, thymus, and intestine [23,24]. Analysis of adult zebrafish tissue identified broad expression of *socs4b*, with higher levels in the brain, which was also previously observed for *socs4a* [36].

To investigate the in vivo function of *socs4b*, zinc-finger nuclease genome-editing technology was employed to mutate the gene. This resulted in the production of a mutant *socs4b* allele, termed *socs4bΔ18*, with an 18 bp deletion that removed the native *socs4b* start codon. RT-PCR analysis confirmed the mutation was present in the transcribed mRNA, which was expressed at similar levels to the wild-type, with no significant impact on the expression of other related *socs* genes. In the absence of commercially available antibodies against the zebrafish Socs4b protein, attempts were made to generate appropriate antibodies in-house, but without success. However, in vitro transcription/translation demonstrated production of a full-length Socs4b protein from the WT sequence and confirmed the absence of full-length Socs4b from the *socs4bΔ18* allele, with low levels of a truncated Socs4b observed, consistent with weak utilization of the next in-frame ATG. This would result in a protein missing the N-terminal 130 amino acids, which includes most of the NTCR4/5 sequences. This suggests that the *socs4bΔ18* allele is likely to be hypomorphic rather than a complete knockout.

Generation of the *socs4bΔ18* allele allowed the analysis of potential in vivo functions. This allele was inherited in a Mendelian manner, with homozygotes showing no signs of disease. External monitoring of homozygote mutant embryos by light microscopy revealed no overt abnormalities during development, or of specific populations, including *isl1a*+ neuronal cells, *ins+* cells of the endocrine pancreas, and MitoTracker-positive lateral neuromasts revealing no obvious changes, which was confirmed with several other markers. Previous research has highlighted the potential involvement of mammalian SOCS4 in wound healing [30] and primordial follicle formation [31]. However, no differences were observed in recovery following fin clipping or in fecundity, suggesting any impacts are relatively minor in zebrafish. These results are consistent with *Socs4* knockout mice, which displayed normal development [24]. This contrasted with the *socs4a* gene, the knockdown of which resulted in decreased *isl1a* expression and induced ectopic expression of *ins* and impacted the formation of a functional sensory system and development of the notochord [36]. Since the Socs4a shows less conservation than Socs4b to the mammalian SOCS4 protein (Supplementary Figure S5), it seems likely that *socs4a* has diverged in function, which is a common occurrence in gene duplicates [48]. A missense mutation in the human *SOCS4* gene has been associated with autoimmunity [29], with *Socs4* knockout mice showing altered viral immunity being associated with enhanced inflammation and pathology [24,26]. However, no alteration was observed with marker genes for leukocytes or T cells *socs4bΔ18* mutants. There was no evidence for compensation by related *socs4a*, *socs5a* or *socs5b* genes, as determined by qRT-PCR, and confirmed by WISH for *socs4a*.

Other studies have suggested that SOCS4 plays a role in the regulation of EGFR signaling, by binding with high affinity to the same EGFR phosphotyrosine site as STAT3, a known downstream signaling molecule [22]. This prompted in vitro investigation of the effects of Socs4b on EGFR signaling. Stimulation with EGF resulted in enhanced levels of pEGFR and pERK in cells transfected with Socs4b WT, but no change in those transfected with Socs4bΔ18. This suggests that zebrafish Socs4b WT can also regulate EGFR signaling, but not the truncated form.

## 5. Conclusions

Zebrafish *socs4b* shows broad expression, but is dispensable for development. However, the encoded Socs4b protein may be involved in EGFR signaling.

## Data Availability

All data analyzed during this study are included in the published article (and associated Supplementary Materials) or are available upon request.

## Supplementary Materials

The following supporting information can be downloaded at: https://www.mdpi.com/article/10.3390/biom14091063/s1, Figure S1: Targeting of the zebrafish socs4b gene using zinc finger nucleases, Figure S2: In vitro transcription/translation analysis, Figure S3: Analysis of mutant socs4b ∆18 gene and its transcript, Figure S4: Impact of zebrafish Socs4b on EGFR signaling, Figure S5: Alignment of SOCS4 proteins.

## References

[1] Yoshimura A., Ito M., Mise-Omata S., Ando M. (2021). SOCS: Negative regulators of cytokine signaling for immune tolerance. Int. Immunol..

[2] Linossi E.M., Nicholson S.E. (2015). Kinase inhibition, competitive binding and proteasomal degradation: Resolving the molecular function of the suppressor of cytokine signaling (SOCS) proteins. Immunol. Rev..

[3] Liongue C., O’Sullivan L.A., Trengove M.C., Ward A.C. (2012). Evolution of JAK-STAT pathway components: Mechanisms and role in immune system development. PLoS ONE.

[4] Sobah M.L., Liongue C., Ward A.C. (2021). SOCS proteins in immunity, inflammatory diseases and immune-related cancer. Front. Med..

[5] Alexander W.S., Starr R., Fenner J.E., Scott C.L., Handman E., Sprigg N.S., Corbin J.E., Cornish A.L., Darwiche R., Owczarek C.M. (1999). SOCS1 is a critical inhibitor of interferon gamma signaling and prevents the potentially fatal neonatal actions of this cytokine. Cell.

[6] Greenhalgh C.J., Rico-Bautista E., Lorentzon M., Thaus A.L., Morgan P.O., Willson T.A., Zervoudakis P., Metcalf D., Street I., Nicola N.A. (2005). SOCS2 negatively regulates growth hormone action in vitro and in vivo. J. Clin. Investig..

[7] Val C.H., de Oliveira M.C., Lacerda D.R., Barroso A., Batista N.V., Menezes-Garcia Z., de Assis D.R.R., Cramer A.T., Brant F., Teixeira M.M. (2020). SOCS2 modulates adipose tissue inflammation and expansion in mice. J. Nutr. Biochem..

[8] Croker B.A., Krebs D.L., Zhang J.G., Wormald S., Willson T.A., Stanley E.G., Robb L., Greenhalgh C.J., Forster I., Clausen B.E. (2003). SOCS3 negatively regulates IL-6 signaling in vivo. Nat. Immunol..

[9] Croker B.A., Metcalf D., Robb L., Wei W., Mifsud S., DiRago L., Cluse L.A., Sutherland K.D., Hartley L., Williams E. (2004). SOCS3 is a critical physiological negative regulator of G-CSF signaling and emergency granulopoiesis. Immunity.

[10] Sobah M.L., Scott A.C., Laird M., Koole C., Liongue C., Ward A.C. (2023). Socs3b regulates the development and function of innate immune cells in zebrafish. Front. Immunol..

[11] Takahashi Y., Carpino N., Cross J.C., Torres M., Parganas E., Ihle J.N. (2003). SOCS3: An essential regulator of LIF receptor signaling in trophoblast giant cell differentiation. EMBO J..

[12] Mori H., Hanada R., Hanada T., Aki D., Mashima R., Nishinakamura H., Torisu T., Chien K.R., Yasukawa H., Yoshimura A. (2004). Socs3 deficiency in the brain elevates leptin sensitivity and confers resistance to diet-induced obesity. Nat. Med..

[13] Yang X.O., Zhang H., Kim B.S., Niu X., Peng J., Chen Y., Kerketta R., Lee Y.H., Chang S.H., Corry D.B. (2013). The signaling suppressor CIS controls proallergic T cell development and allergic airway inflammation. Nat. Immunol..

[14] Palmer D.C., Guittard G.C., Franco Z., Crompton J.G., Eil R.L., Patel S.J., Ji Y., Van Panhuys N., Klebanoff C.A., Sukumar M. (2015). Cish actively silences TCR signaling in CD8+ T cells to maintain tumor tolerance. J. Exp. Med..

[15] Delconte R.B., Guittard G., Goh W., Hediyeh-Zadeh S., Hennessy R.J., Rautela J., Davis M.J., Souza-Fonseca-Guimaraes F., Nunes J.A., Huntington N.D. (2020). NK cell priming from endogenous homeostatic signals is modulated by CIS. Front. Immunol..

[16] Louis C., Souza-Fonseca-Guimaraes F., Yang Y., D’Silva D., Kratina T., Dagley L., Hediyeh-Zadeh S., Rautela J., Masters S.L., Davis M.J. (2020). NK cell-derived GM-CSF potentiates inflammatory arthritis and is negatively regulated by CIS. J. Exp. Med..

[17] Maymand S., Lakkavaram A.L., Naser W., Rasighaemi P., Dlugolenski D., Liongue C., Stambas J., de Koning-Ward T.F., Ward A.C. (2023). Role of cytokine-inducible SH2 domain-containing (CISH) protein in the regulation of erythropoiesis. Biomolecules.

[18] Naser W., Maymand S., Rivera L.R., Connor T., Liongue C., Smith C.M., Aston-Mourney K., McCulloch D.R., McGee S.L., Ward A.C. (2022). Cytokine-inducible SH2 domain containing protein contributes to regulation of adiposity, food intake, and glucose metabolism. FASEB J..

[19] Wang B., Wangkahart E., Secombes C.J., Wang T. (2019). Insights into the evolution of the suppressors of cytokine signaling (SOCS) gene family in vertebrates. Mol. Biol. Evol..

[20] Krebs D.L., Uren R.T., Metcalf D., Rakar S., Zhang J.G., Starr R., De Souza D.P., Hanzinikolas K., Eyles J., Connolly L.M. (2002). SOCS-6 binds to insulin receptor substrate 4, and mice lacking the SOCS-6 gene exhibit mild growth retardation. Mol. Cell. Biol..

[21] Lawrenson I.D., Krebs D.L., Linossi E.M., Zhang J.G., McLennan T.J., Collin C., McRae H.M., Kolesnik T.B., Koh K., Britto J.M. (2017). Cortical layer inversion and deregulation of Reelin signaling in the absence of SOCS6 and SOCS7. Cereb. Cortex.

[22] Kario E., Marmor M.D., Adamsky K., Citri A., Amit I., Amariglio N., Rechavi G., Yarden Y. (2005). Suppressors of cytokine signaling 4 and 5 regulate epidermal growth factor receptor signaling. J. Biol. Chem..

[23] Delgado-Ortega M., Melo S., Meurens F. (2011). Expression of SOCS1-7 and CIS mRNA in porcine tissues. Vet. Immunol. Immunopathol..

[24] Kedzierski L., Linossi E.M., Kolesnik T.B., Day E.B., Bird N.L., Kile B.T., Belz G.T., Metcalf D., Nicola N.A., Kedzierska K. (2014). Suppressor of cytokine signaling 4 (SOCS4) protects against severe cytokine storm and enhances viral clearance during influenza infection. PLoS Pathog..

[25] Hu G., Zhou R., Liu J., Gong A.Y., Chen X.M. (2010). MicroRNA-98 and let-7 regulate expression of suppressor of cytokine signaling 4 in biliary epithelial cells in response to *Cryptosporidium parvum* infection. J. Infect. Dis..

[26] Kedzierski L., Er Qi Tan A., Jia Hui Foo I., Narayanan D., Moily N., McQuilten H.A., Nicholson S.E., Fazakerley J.K. (2023). In Semliki Forest virus encephalitis, suppressor of cytokine signaling 4 (SOCS4) is an essential modulator of immune responses that mediates the balance between immunopathology and virus clearance. Immunol. Cell Biol..

[27] Brender C., Columbus R., Metcalf D., Handman E., Starr R., Huntington N., Tarlinton D., Odum N., Nicholson S.E., Nicola N.A. (2004). SOCS5 is expressed in primary B and T lymphoid cells but is dispensable for lymphocyte production and function. Mol. Cell. Biol..

[28] Kedzierski L., Tan A.E.Q., Foo I.J.H., Nicholson S.E., Fazakerley J.K. (2022). Suppressor of cytokine signalling 5 (SOCS5) modulates inflammatory responses during alphavirus infection. Viruses.

[29] Arts P., Plantinga T.S., van den Berg J.M., Gilissen C., Veltman J.A., van Trotsenburg A.S., van de Veerdonk F.L., Kuijpers T.W., Hoischen A., Netea M.G. (2015). A missense mutation underlies defective SOCS4 function in a family with autoimmunity. J. Intern. Med..

[30] Feng Y., Sanders A.J., Morgan L.D., Owen S., Ruge F., Harding K.G., Jiang W.G. (2017). In vitro significance of SOCS-3 and SOCS-4 and potential mechanistic links to wound healing. Sci. Rep..

[31] Sutherland J.M., Keightley R.A., Nixon B., Roman S.D., Robker R.L., Russell D.L., McLaughlin E.A. (2012). Suppressor of cytokine signaling 4 (SOCS4): Moderator of ovarian primordial follicle activation. J. Cell. Physiol..

[32] Choi T.Y., Choi T.I., Lee Y.R., Choe S.K., Kim C.H. (2021). Zebrafish as an animal model for biomedical research. Exp. Mol. Med..

[33] O’Sullivan L.A., Noor S.M., Trengove M.C., Lewis R.S., Liongue C., Sprigg N.S., Nicholson S.E., Ward A.C. (2011). Suppressor of cytokine signaling 1 regulates embryonic myelopoiesis independently of its effects on T cell development. J. Immunol..

[34] Dai Z., Wang H., Jin X., Wang H., He J., Liu M., Yin Z., Sun Y., Lou Q. (2015). Depletion of suppressor of cytokine signaling-1a causes hepatic steatosis and insulin resistance in zebrafish. Am. J. Physiol. Endocrinol. Metab..

[35] Lewis R.S., Noor S.M., Fraser F.W., Sertori R., Liongue C., Ward A.C. (2014). Regulation of embryonic hematopoiesis by a cytokine-inducible SH2 domain homolog in zebrafish. J. Immunol..

[36] Trengove M., Wyett R., Liongue C., Ward A.C. (2022). Functional analysis of zebrafish socs4a: Impacts on the notochord and sensory function. Brain Sci..

[37] Thisse C., Thisse B. (2008). High-resolution in situ hybridization to whole-mount zebrafish embryos. Nat. Protoc..

[38] Livak K.J., Schmittgen T.D. (2001). Analysis of relative gene expression data using real-time quantitative PCR and the 2−ΔΔCT method. Methods.

[39] Urnov F.D., Rebar E.J., Holmes M.C., Zhang H.S., Gregory P.D. (2010). Genome editing with engineered zinc finger nucleases. Nat. Rev. Genet..

[40] Kulinski J., Besack D., Oleykowski C.A., Godwin A.K., Yeung A.T. (2000). CEL I enzymatic mutation detection assay. Biotechniques.

[41] Papasani M.R., Robison B.D., Hardy R.W., Hill R.A. (2006). Early developmental expression of two insulins in zebrafish (*Danio rerio*). Physiol. Genom..

[42] Inoue A., Takahashi M., Hatta K., Hotta Y., Okamoto H. (1994). Developmental regulation of islet-1 mRNA expression during neuronal differentiation in embryonic zebrafish. Dev. Dyn..

[43] Liu F., Wen Z. (2002). Cloning and expression pattern of the lysozyme C gene in zebrafish. Mech. Dev..

[44] Willett C.E., Cherry J.J., Steiner L. (1997). Characterization and expression of the recombination activating genes (rag1 and rag2) of zebrafish. Immunogenetics.

[45] Thomas E.D., Cruz I.A., Hailey D.W., Raible D.W. (2015). There and back again: Development and regeneration of the zebrafish lateral line system. Wiley Interdiscip. Rev. Dev. Biol..

[46] Bullock A.N., Rodriguez M.C., Debreczeni J.E., Songyang Z., Knapp S. (2007). Structure of the SOCS4-ElonginB/C complex reveals a distinct SOCS box interface and the molecular basis for SOCS-dependent EGFR degradation. Structure.

[47] Oates A.C., Wollberg P., Pratt S.J., Paw B.H., Johnson S.L., Ho R.K., Postlethwait J.H., Zon L.I., Wilks A.F. (1999). Zebrafish stat3 is expressed in restricted tissues during embryogenesis and stat1 rescues cytokine signaling in a STAT1-deficient human cell line. Dev. Dyn..

[48] Kassahn K.S., Dang V.T., Wilkins S.J., Perkins A.C., Ragan M.A. (2009). Evolution of gene function and regulatory control after whole-genome duplication: Comparative analyses in vertebrates. Genome Res..

