# Supplementary material for: Zebrafish Suppressor of Cytokine Signaling 4b (Socs4b) Is Dispensable for Development but May Regulate Epidermal Growth Factor Receptor Signaling
Source: Biomolecules. 2024 Aug 26;14(9):1063. doi: 10.3390/biom14091063 (PMC11430285; doi:10.3390/biom14091063)

Figure S1. Targeting of the zebrafish *socs4b* gene using zinc finger nucleases. Schematic representation of: (A) the *socs4b* gene (GenBank NC_0071267.7; GRCz11 Chr15), with exons shown as black boxes and introns as black lines; (B) the corresponding mRNA (GenBank XM_005173430.4) with the coding sequence shaded; (C) the encoded Socs4b protein (GenBank NP_001107273.1), with the NTCR4-5, SH2 and SOCS box domains indicated based on. The site targeted by zinc finger nucleases and the locations of primers used in genotyping are indicated in panel A with scissors and blue arrows, respectively, with primers used for RT-PCR shown in panel B with green and brown arrows. The potential protein produced by the *socs4b Δ18* allele is displayed in panel C.

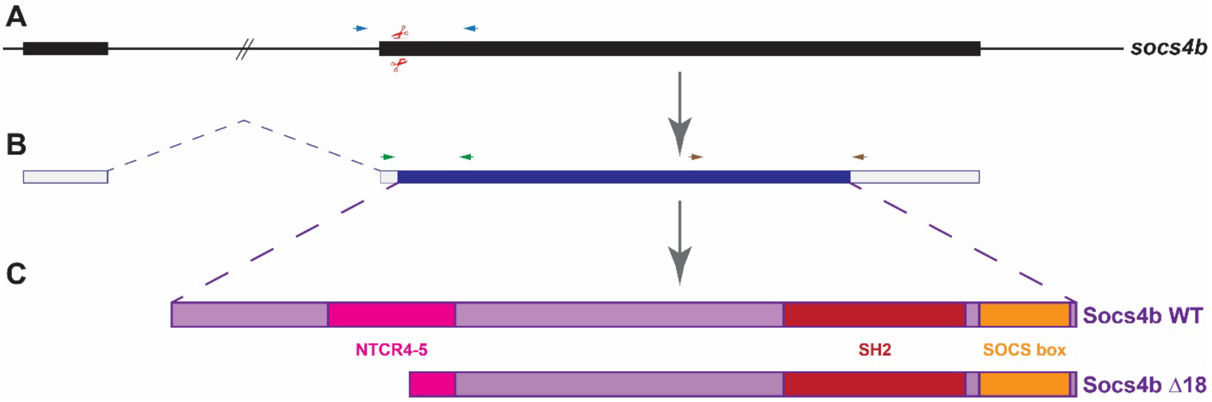

Figure S2. In vitro transcription/translation analysis. Original blots for in vitro transcription/translation performed on Socs4b WT, Socs4bΔ18 and no template control (NTC) as indicated used to make Figure 2C.

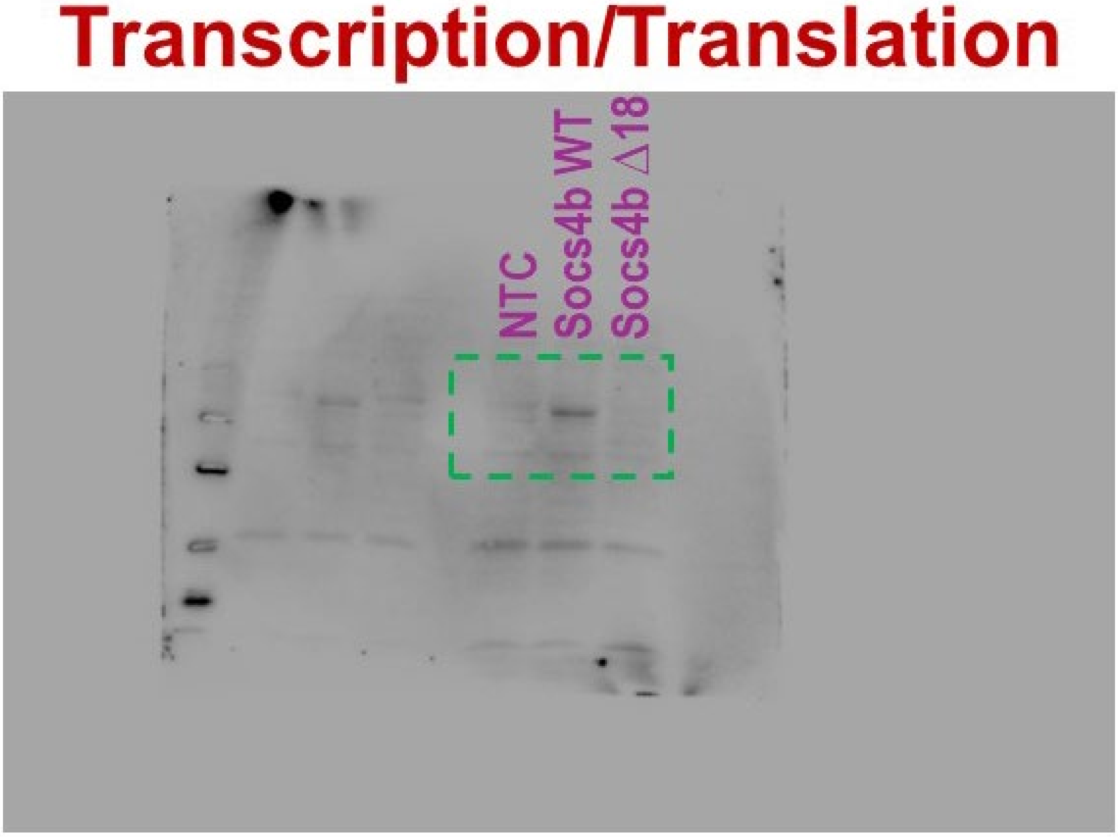

Figure S3. Analysis of mutant *socs4b Δ18* gene and its transcript. PCR of genomic DNA from homozygous wild-type *socs4b* (+/+), heterozygous (+/Δ) and homozygous (Δ/Δ) *socs4b Δ18* progeny with primers spanning the mutation site, showing wild-type product with a blue arrow and the 18 bp shorter mutant product with a red arrow (A). RT-PCR of total RNA extracted from +/+, +/Δ and Δ/Δ embryos of the indicated age using primers specific to regions of *socs4* mRNA encoding the Socs4b N-terminus (N) and C-terminus (C) along with those to *actb* (encoding β-actin) as a control, showing results for samples (s) and RT-negative controls (c) (B). The wild-type product using the N-terminal primers is indicated with a blue arrow and the shorter mutant product with a red arrow, with the equivalent products obtained using the C-terminal primers is shown with a green arrow.

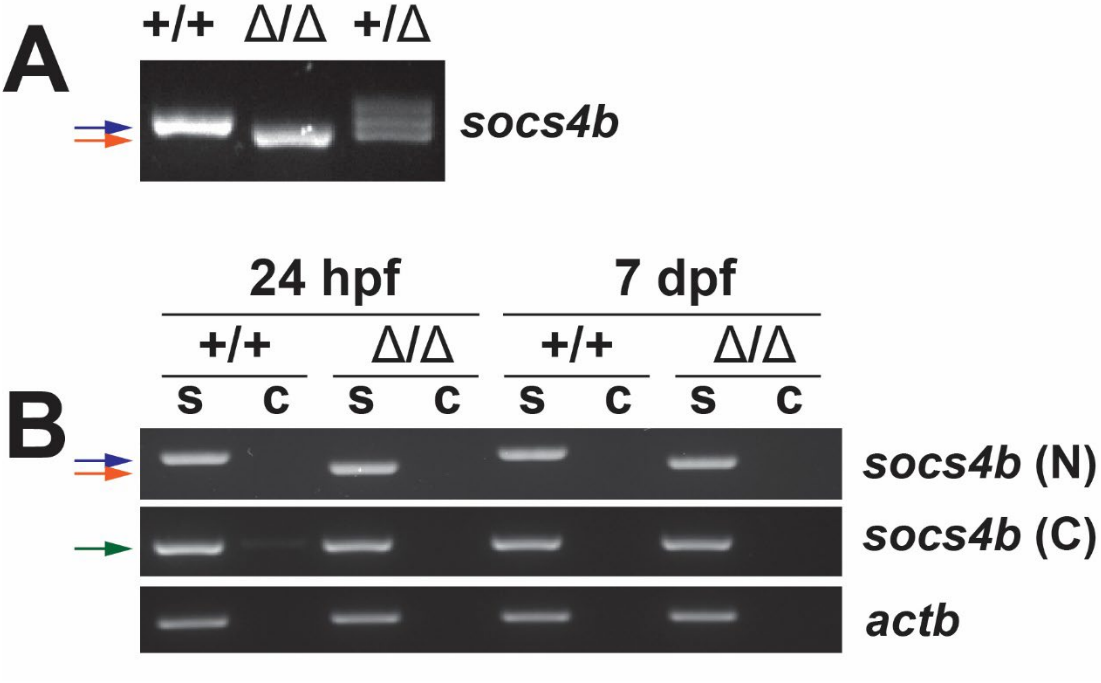

Figure S4. Impact of zebrafish Socs4b on EGFR signaling. Original blots for HEK293T cells transfected with empty vector control or plasmids encoding Flag-tagged Socs4b WT or Socs4b Δ18 stimulated with EGF for the time shown (min) subjected to Western antibodies against Flag, pEGFR, pERK, tERK and GAPDH as indicated used to make panels for Figure 4.

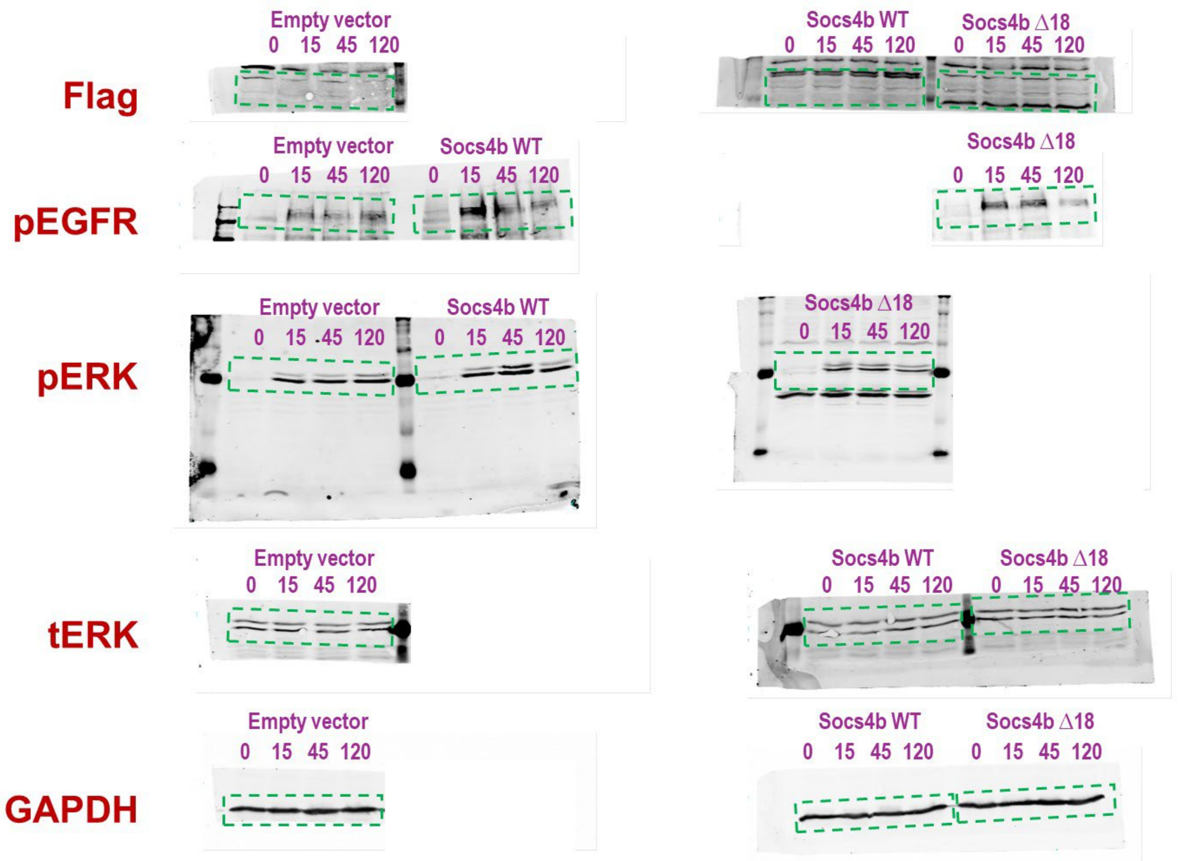

Figure S5. Alignment of SOCS4 proteins. Alignment of human (hs: *Homo sapiens*) SOCS4 with zebrafish (dr: *Danio rerio*) Socs4a and Socs4b. Domains are shown, including the N-terminal conserved region 4-5 (NTCR4-5), as well as the Src-homology 2 (SH2) and SOCS box domains. Asterisks (*) represent identical residues, colons (:) represent highly related residues and periods (.) represent moderately related residues.

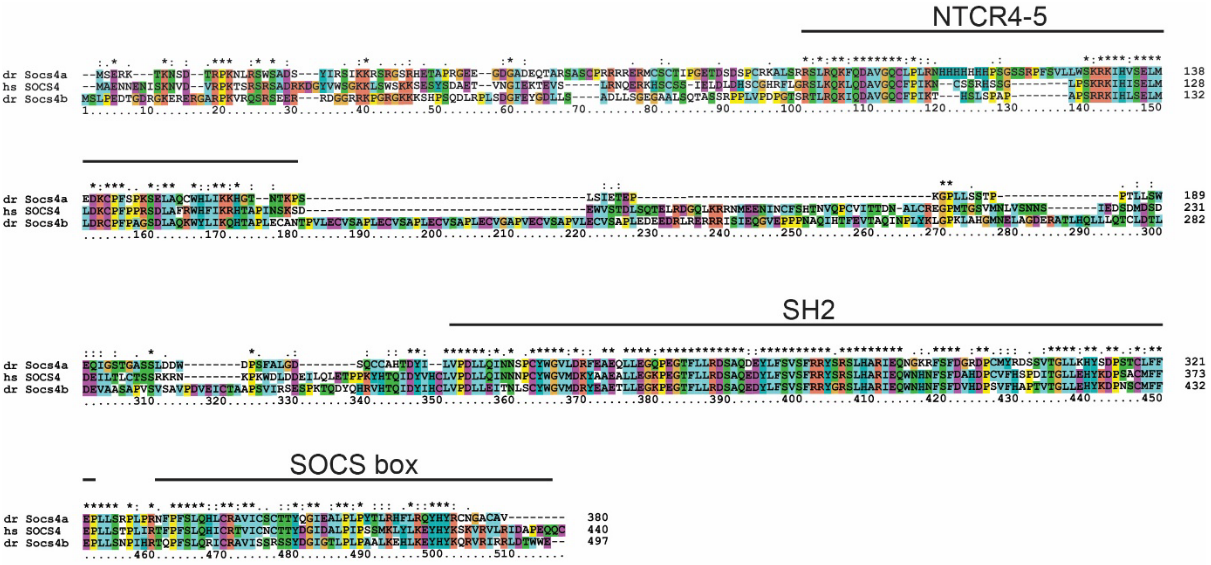

Supplement: Supplementary file 1 [file biomolecules-14-01063-s001.zip › biomolecules-3114403-supplementary.pdf]
